# Genome-wide DNA Methylation and RNAseq Analyses Identify Aberrant Signalling Pathways in Focal Cortical Dysplasia (FCD) Type II

**DOI:** 10.1038/s41598-018-35892-5

**Published:** 2018-12-19

**Authors:** Aparna Banerjee Dixit, Devina Sharma, Manjari Tripathi, Arpna Srivastava, Debasmita Paul, Deepak Prakash, Chitra Sarkar, Krishan Kumar, Jyotirmoy Banerjee, P. Sarat Chandra

**Affiliations:** 1 Center of Excellence for Epilepsy, A joint NBRC-AIIMS collaboration, NBRC, Manesar, India; 2 Dr. B R Ambedkar Center for Biomedical Research, University of Delhi, Delhi, India; 3 Department of Neurology, AIIMS, New Delhi, India; 4 Department of Neurosurgery, AIIMS, New Delhi, India; 5 Department of Forensic Medicine and Toxicology, AIIMS, New Delhi, India; 6 Department of Pathology, AIIMS, New Delhi, India; 7 Department of Biophysics, AIIMS, New Delhi, India

## Abstract

Focal cortical dysplasia (FCD) is one of the most common pathologies associated with drug-resistant epilepsy (DRE). The pharmacological targets remain obscured, as the molecular mechanisms underlying FCD are unclear. Implications of epigenetically modulated aberrant gene expression in disease progression are reported in various DRE pathologies except FCD. Here we performed genome-wide CpG-DNA methylation profiling by methylated DNA immunoprecipitation (MeDIP) microarray and RNA sequencing (RNAseq) on cortical tissues resected from FCD type II patients. A total of 19088 sites showed altered DNA methylation in all the CpG islands. Of these, 5725 sites were present in the promoter regions, of which 176 genes showed an inverse correlation between methylation and gene expression. Many of these 176 genes were found to belong to a cohesive network of physically interacting proteins linked to several cellular functions. Pathway analysis revealed significant enrichment of receptor tyrosine kinases (RTK), EGFR, PDGFRA, NTRK3, and mTOR signalling pathways. This is the first study that investigates the epigenetic signature associated with FCD type II pathology. The candidate genes and pathways identified in this study may play a crucial role in the regulation of the pathogenic mechanisms of epileptogenesis associated with FCD type II pathologies.

## Introduction

Focal cortical dysplasia (FCD) is a common pathology associated with drug-resistant epilepsy (DRE) caused by the malformations of cortical development (MCDs) and accounts for ~30% of the cases referred to surgery, however, 20–60% of this specific subgroup of patients is not seizure free even after the resective surgery^1,2^.

The International League Against Epilepsy (ILAE) classification of FCDs: type I, type II and type III, describes distinct subtypes with different clinical presentations, topographic localization, and response to surgery^1^. FCD type II, a more homogeneous malformation with well-described histopathological features is particularly frequent in frontal and parietal lobes, and can present as either small or almost invisible bottom of sulcus dysplasia or larger dysplastic regions affecting more than a single gyrus. FCD type II is characterized by malformations resulting from disrupted cortical lamination and specific cytological abnormalities - type IIa with dysmorphic neurons and type IIb with dysmorphic neurons and balloon cells^3^. Other than these cytological differences, no subtype-specific clinical and imaging findings have been observed for FCD type IIa and IIb pathologies^4^. Aberrant cortical development at the level of neuronal-glial proliferation and faulty differentiation during migration of neurons leads to the occurrence of abnormal cells in FCD^1^. Genetic, epigenetic, and environmental factors cumulatively may play a crucial role in MCD^5^.

In the past two decades, aberrant gene expression has been reported in different epilepsy pathologies^6,7^. Numerous genes associated with MCD with no family pedigree (e.g. *TSC1, TSC2, LIS-1, DCX, FLN1, CNTNAP2, SCN1A, STXBP1,* and *FLN1*) have been reported^1,3^. Overexpression of neurotrophin receptors TrkB and TrkC with a potential role in epileptogenesis was demonstrated in dysmorphic neurons of FCD^6,8^. Altered expression and co-assembly of NR2B, GluN2A, and GluN2B type N-methyl-D-aspartate (NMDA) receptor subunits, upregulation of the GluN1 subunit, GluA2/3, α-amino-3-hydroxy-5-methyl-4-isoxazolepropionic acid (AMPA) receptor subunits, metabotropic glutamate receptors mGluR1 and mGluR5, and chloride transporters NKCC1 and KCC2 were reported in dysplastic cortical regions^3,6,8,9^. Abnormal neuronal and glial differentiation, cell immaturity, and cytoskeletal abnormalities have been reported in both FCD type IIa and IIb pathologies^4^. Dysmaturity hypothesis supported by the presence of potentially excitatory GABA-mediated neurotransmission and expression of early progenitor cell markers including nestin, vimentin, Pax6, CD133, CD34, GFAP, and MAP2 is so far the most commonly accepted hypothesis^3,6,10^. Recent evidences suggest a major role of mTOR pathway in the pathogenesis of both FCD type IIa and IIB including other related MCD^3,11–15^. Despite considerable progress in understanding of the molecular mechanisms underlying FCDs, till date, there are no molecular biomarkers available to classify the subtype, define the epileptogenic zone (EZ), predict the surgical outcome, or determine drug resistance in patients.

Epigenetic modifications including DNA methylation can be an important regulator of gene expression changes associated with epilepsy^16^. Previously, genome-wide aberrant promoter methylation, and association of increased Reelin promoter methylation with granule cell dispersion in human temporal lobe epilepsy (TLE) has been reported^17,18^. Increased DNA methyltransferase (*DNMT*) gene expression is reported in human TLE and inhibition of DNMTs in animal models was shown to reduce DNA methylation and epileptogenesis^19,20^. Miller-Delaney *et al*. have described differential DNA methylation in defining status epilepticus (SE) and epileptic tolerance^21^. More recent studies have reported genome-wide DNA methylation changes in tissues as well as blood samples in human epilepsies including MTLE^22,23^.

However, till date there is no report on genome-wide DNA methylation and transcriptome analysis of tissues resected from FCD patients. In the present study, we have identified and discussed, for the first time, the role of epigenetically deregulated genes and pathways with a potential role in the pathophysiology of FCD type II patients.

## Results

### Differential DNA methylation in FCD

**Figure 1:**
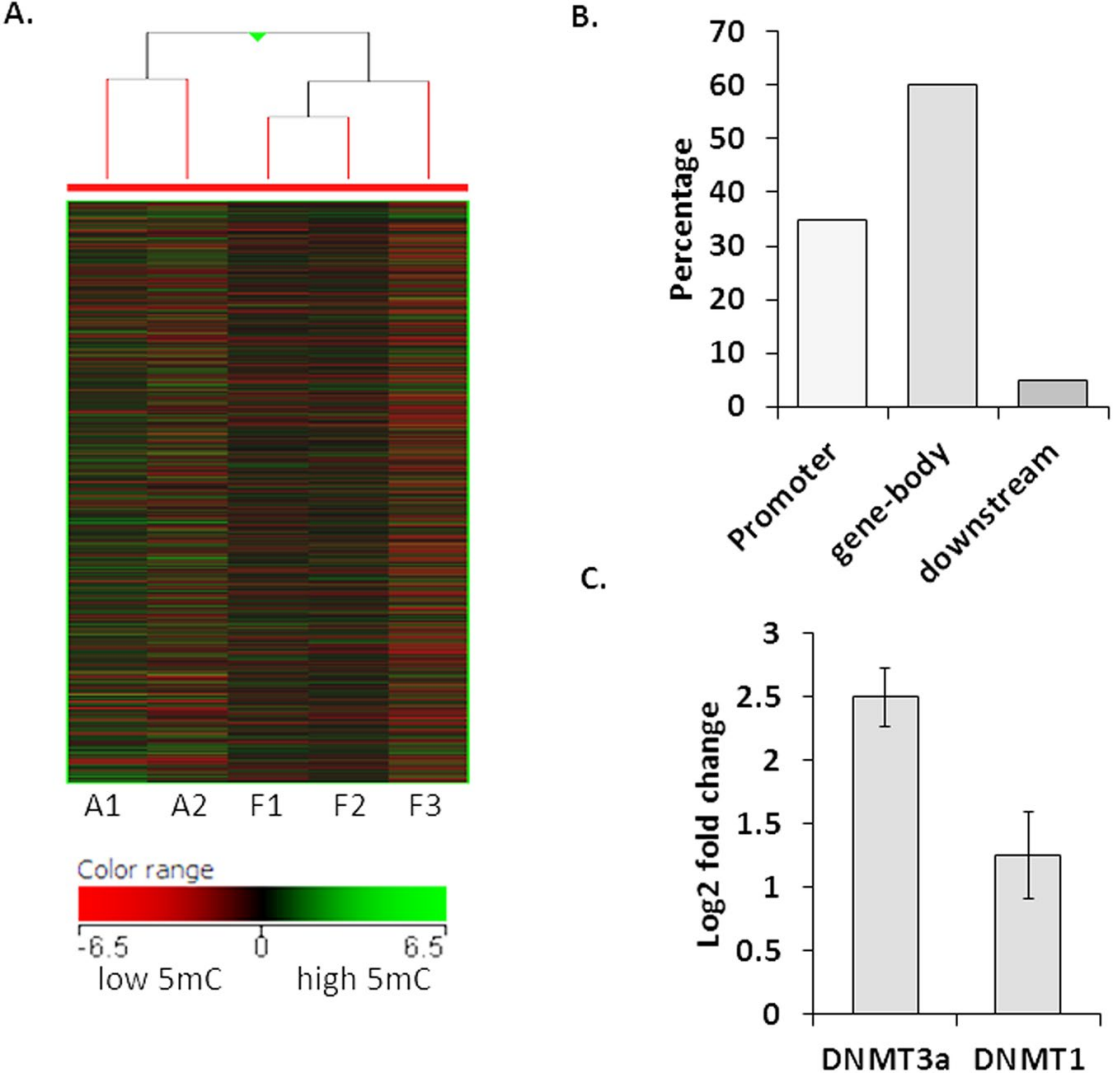
Genomic distribution of methylation changes and qPCR analysis of DNMTs in brain tissues resected from FCD type II patients. (**A**) Heatmap depicting Hierarchical clustering ofall samples and genomic regions according to differential methylation profiles, green methylation up (high 5mC), red methylation down (low 5mC). Clustering was done using the Hierarchical condition package in the GeneSpring GX software (version 13.0). (**B**) Bar chart showing genomic distribution of CpG sites with altered DNA methylation patterns in patients with FCD type II as compared to the autopsy controls. (**C**) mRNA levels of *DNMT3α* showing increased expression whereas mRNA levels of *DNMT1* remain unaltered. Relative changes in gene expression were calculated using the ΔΔCq method with *HPRT* as a reference gene. Mean increase in transcript levels was statistically significant (*p < 0.05) with respect to the controls. Data represent mean ± SEM (n = 10), each done in triplicate.

To examine the role of DNA methylation in FCD type II patients, DNA methylation microarray analysis of tissues resected from patients (n = 3) was carried out and compared with autopsy controls (n = 2). Pairwise comparison of global DNA methylation detected strong differences in methylation patterns between controls and FCD samples. Hierarchical cluster analysis was used to assess and visualize underlying differences between samples and phenotypes. As shown in Fig. 1A, DNA methylation profiles readily discriminated samples and controls into discrete groups. There were a total of 12686 hypermethylated and 6402 hypomethylated sites with adjusted *p-*values < 0.05 and fold-change (FC) ≥ 2 (Supplementary Table S1). Out of these, 3656 genes were hypermethylated and 2069 were hypomethylated in the promoter regions. The distribution and location of the CpG sites with altered methylation are shown in Fig. 1B. Nearly 35% of the differentially methylated sites were either located on or were present in the vicinity of the promoter regions, suggesting methylation as a regulatory mechanism for gene expression regulation. A high percentage (60%) of altered methylation sites were clustered within the gene body and only 5% were found to be in present in the downstream regions (Fig. 1B). Since differential methylation of CpG-rich promoters is well accepted as an indicator of gene expression changes in physiologic and pathologic conditions, we filtered the gene list and selected those showing differential methylated promoters for further analysis. Enrichment of MeDIP-DNA was confirmed by a qPCR-based assay. The fold-enrichment for *H19* compared with *GAPDH* determined using the comparative Ct method, ranged from 2- to 10-fold for each of the samples tested (data not shown). In order to correlate the altered DNA methylation with the levels of DNMTs, we determined the levels of *DNMT3α* and *DNMT1* in the same samples and found that while *DNMT3α* expression levels were significantly upregulated (*p* < 0.05) in patients, *DNMT1* mRNA levels were unchanged (Fig. 1C).

### Differential gene expression in FCD

We examined the gene expression from the same tissue specimens to analyze the biological relevance of methylation changes. RNA from all the samples (both FCD and autopsy) used in this study had RIN values more than 6 (range 6.1–8.2; mean 7.15) confirming that the RNA samples were of excellent quality^6^. Supplementary Table S2 provides a summary of the RNAseq data of the five tissue samples, 3 FCD type II (F1-F3) and 2 autopsy (A1-A2) controls included in the study. In total, we obtained 56 M, 64 M and 34 M, 38 M, and 32 M sequence reads for the samples A1, A2, F1, F2, and F3, respectively. Low-quality bases and reads were filtered out from the datasets prior to read mapping. 42 M, 52 M, 23 M, 25 M, and 23 M reads were mapped to human genome for A1, A2, F1, F2, and F3 samples, respectively. DGE analysis revealed upregulation of 386 and downregulation of 220 genes in FCD patients with log2 FC ≥ 2 and *q*-value < 0.05 when compared with the controls (Supplementary Table S2).

### Inverse correlation between promoter methylation and gene expression in FCD

**Figure 2:**
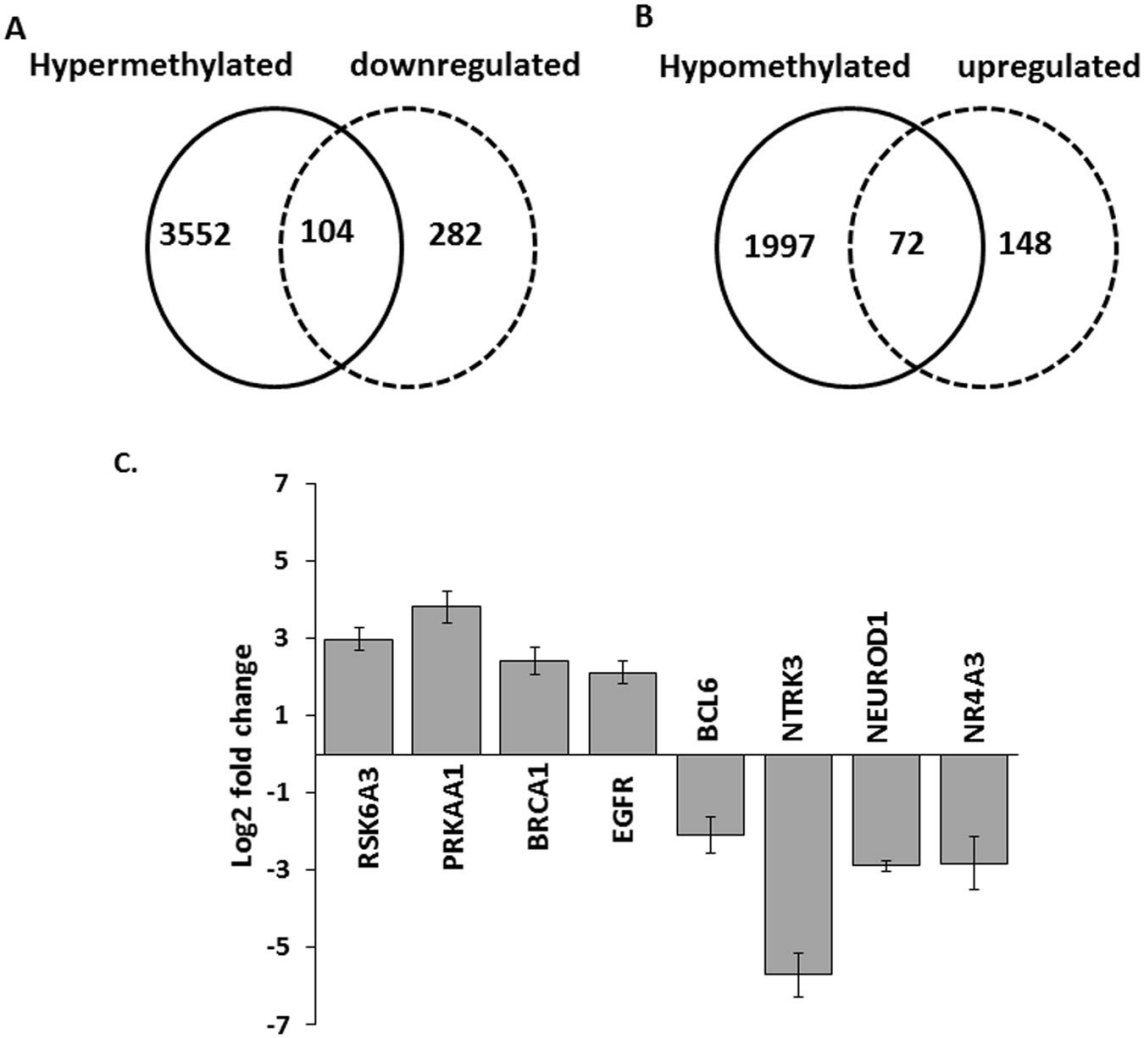
Integrative analysis of genes with differentially methylated promoters and differential expression and qPCR analysis of selected genes in brain tissues resected from FCD type II patients. (**A,B**) Venn diagram showing total number of hypermethylated/downregulated (A; left panel) and hypomethylated/upregulated genes (B; right panel) genes. A total of 104 genes were hypermethylated and downregulated in expression and 72 genes were hypomethylated and upregulated in their expression. (**C**) The expression level of upregulated mRNAs (*RPS6KA3, PRKAA1, BRCA1*, and *EGFR*) and downregulated mRNAs (*BCL6, NTRK3, NEUROD1*, and *NR4A3*) as identified in the RNAseq data were further validated by real-time PCR. Relative changes in gene expression were calculated using the ΔΔCq method with *HPRT* as a reference gene. Mean increases in transcript levels were statistically significant (*p < 0.05) with respect to control. Data represent mean ± SEM (n = 10), each done in triplicate.

To determine whether DNA methylation changes associated with the promoter regions targeted gene expression, we did an integrative analysis of the 5725 genes showing altered methylation in the promoter regions with the 606 DEGs in the same FCD type II patients. 386 DEGs showing upregulation were integrated with the 2069 hypomethylated genes and 220 DEGs showing downregulation were integrated with the 3656 hypermethylated genes using the GeneSpring software. A total of 176 DEGs genes were inversely correlating with the promoter methylation patterns (Supplementary Table S3). Promoters of 104 downregulated DEGs were hypermethylated and 72 upregulated DEGs were hypomethylated **(**Fig. 2A,B**)**, suggesting that abnormal DNA methylation might have functional consequences. Expression profiles of 8 of these filtered 176 genes, i.e., *EGFR, NEUROD1, RPS6KA3, DLG1, NR4A3, NTRK3, BRCA1*, and *BCL6*, were further validated by qPCR in 10 FCD type II patients and 10 control samples (Fig. 2C). Fold-change in the expression of these DEGs, as observed in qPCR, correlated with the RNAseq data.

### Canonical pathways associated with FCD type II

**Figure 3:**
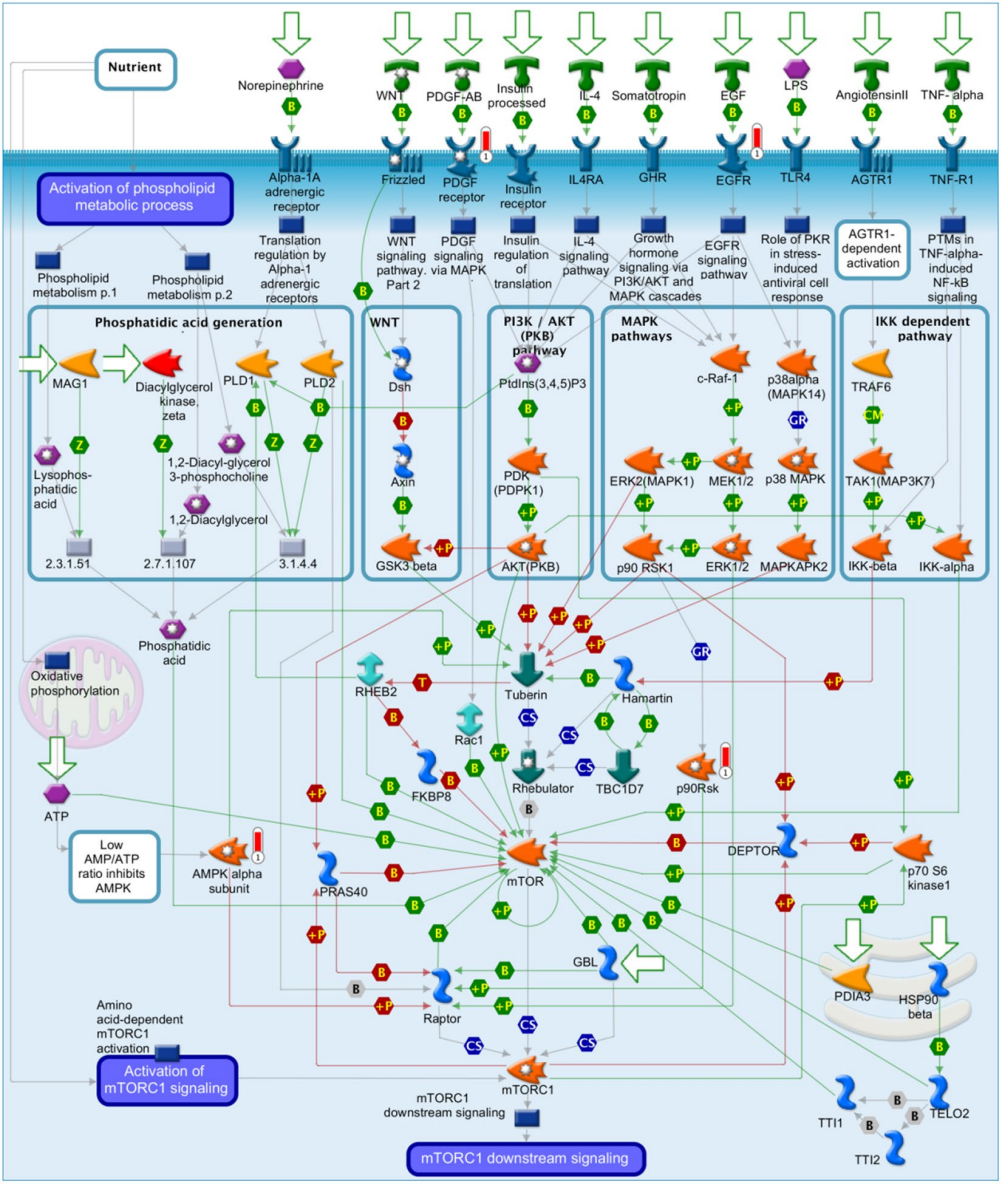
Canonical pathway analysis using MetaCore identified EGFR, PDGFR, and mTOR signalling as the top 3 canonical pathways modulated in FCD type II patients. Red thermometers show an object that is upregulated. The big arrows indicate the “pathway start”. GR: group relation; CS: complex subunit; B: Boxes on lines denote the type of regulation where P is phosphorylation, B is binding and Z is catalysis.

Functional gene clustering of 176 DEGs that showed an inverse correlation in methylation status and gene expression, revealed top 8 enriched clusters based on significant scores **(**Table 1**)**. The biological processes revealed by GO terms in the cluster analysis included regulation of neurogenesis, neural development, transcription, metabolism, cell migration, ion channel activity, and negative regulation of cell death and apoptosis. Previous reports as well as our current data suggest modulation of multiple biological processes, including neuronal network and synaptic activity during epileptogenesis. Pathway analysis of these 176 DEGs using Metaore identified more than 100 pathways, of which top 10 canonical pathways were further analysed for their potential role in FCD **(**Supplementary Fig. S1). The most important pathways identified involved PDGFR, EGFR, and mTOR signalling pathways **(**Fig. 3**)**.

**Table 1:** Enrichment of GO terms using DAVID functional annotation tool of epigenetically modulated gene expression in FCD type II.

Term	Count	p-value	Term	Count	*p*-value
**Cluster 1 Enrichment score- 2.54**			**Cluster 5 Enrichment score- 1.55**		
GO:0050767- regulation of neurogenesis	8	1.5E-3	GO:0046872- metal ion binding	54	2.3E-2
GO:0051960- regulation of nervous system	8	3.4E-3	GO:0043169- cation binding	54	2.8E-2
GO:0060284- regulation of cell development	8	4.9E-3	GO:0043167- ion binding	54	3.6E-2
**Cluster 2 Enrichment score- 2.33**			**Cluster 6 Enrichment score- 1.46**		
GO:0045892-neagtive regulation of transcription	13	2.4E-3	GO:0042981- regulation of apoptotic process	15	3.3E-2
GO:0010629- negative regulation of gene expression	13	5.1E-3	GO:0043067- regulation of programmed cell death	15	3.6E-2
GO:0045934- negative regulation of nucleobase-containing compound metabolic process	13	5.8E-3	GO:0010941- regulation of cell death	15	3.7E-2
GO:0051172- negative regulation of nitrogen compound metabolic process	13	6.4E-3			
**Cluster 3 Enrichment score- 1.98**			**Cluster 7 Enrichment score- 1.37**		
GO:0016477- cell migration	9	7.0E-3	GO:0005524- Protein kinase, ATP binding site	10	3.2E-2
GO:0051674- localization of cell	9	1.3E-2	GO:0004672- Protein kinase core	10	4.0E-2
GO:0048870- cell motility	9	1.3E-2			
**Cluster 4 Enrichment score- 1.98**			**Cluster 8 Enrichment score- 1.09**		
GO:0043066- negative regulation of apoptotic process	10	9.9E-3	GO:0005261- cation channel activity	8	2.1E-2
			GO:0005216- ion channel activity	8	9.6E-2
			GO:0022838- substrate-specific channel activity	8	1.1E-1
GO:0043069- negative regulation of programmed cell death	10	1.1E-2	GO:0015267- channel activity	8	1.2E-1
negative regulation of cell death	10	1.1E-2	GO:0022803- passive transmembrane transporter activity	8	1.2E-1

**Figure 4:**
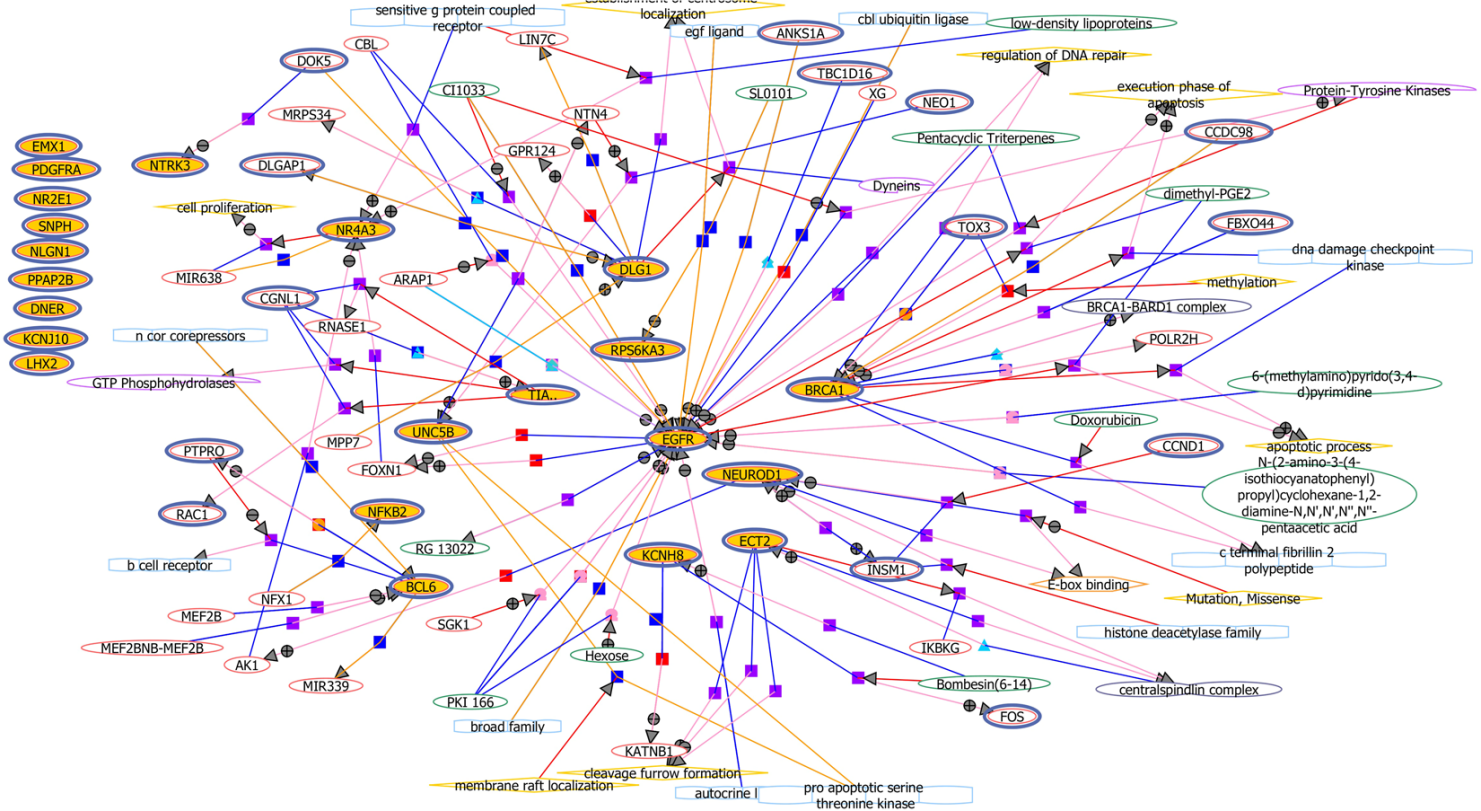
Gene network analysis showing association between significantly modulated genes. Genes showing no association are placed separately from the gene network Different coloured edges with arrows shows the direction of interactions. Circles with + and − symbols represents positive and negative regulations. Symbols (small squares, triangles and circles) shows various modes of regulations like, binding, expression, transport, metabolism etc. Various other entities not reported in our study (small molecules, genes, function, enzymes etc) to which these genes interact are enclosed in different colored shapes. Details of the graphical display of these associations are provided in Supplementary Fig. S3.

Based on the GO enrichment scores and pathway analysis results, a list of 25 genes involved in top 10 canonical pathways was chosen for further network analysis. Out of these 25 potential candidates, 8 genes (*NEUROD1, NCAM-1, EGFR, PDGFRA, UNC5B, KCNJ10, NF-kB2, NR4A3*) have previously been shown to be associated with epilepsy, whereas 17 genes (*PPA2B, ECT2, SNPH, BCL6, DLG1, RPS6KA3, NTRK3, PRKAA1, DNER, NLGN1, TIAM1, KCNH8, EMX1, NKX6, LHX2, NR2E1, BRCA1*) are novel in our study **(**Table 2**)**. Further grouping of the 25 genes was done based on their molecular functions. These groups include genes involved in RTK and mTOR signallings (*EGFR, PDGFRA, NTRK3, RPS6KA3, PRKAA1*), synaptic transmission (*KCNJ10, KCNH8, SNPH, DLG1, PPA2B*), and neuronal development and cell-cell interactions (*EMX1, NKX6-1, LHX2, NEUROD1, NR2E1, NR4A3, TIAM1, ECT2, BCL6, NF-kB2, BRCA1*, *NCAM-1, UNC5B, DNER, NLGN1*) **(**Table 2**)**. Gene network analysis was performed to look at the associations of the filtered 25 genes by indirect interaction analysis. Various genes such as *EGFR, NEUROD1, RPS6KA3, DLG1, NR4A3, BRCA1, KCNH8, UNC5B, BCL6, ECT2, NF-kB2, NTRK3* also showed associations and formed functional networks associated with FCD type II **(**Fig. 4**)**.

**Table 2:** Epigenetically modified DEGs with potential role in FCD type II pathophysiology.

Gene ID^a^	Gene name	Function
**RTK and mTOR signalling**		
EGFR/**R**	Epidermal growth factor receptor	Governs cell fate specification, cell proliferation, migration in the neural stem cell compartment, and glial development
PDGFRA/**R**	Platelet- derived growth factor receptor alpha	Regulation of embryonic development, Cell proliferation, survival, and chemotaxis
NTRK3/**R**	Neurotrophic tyrosine receptor kinase (TRK)	Cell differentiation
RPS6KA3/**N**	Ribosomal S6 kinase 3	mTOR signalling, synaptic plasiticity, ribosome biogenesis, and extraribosomal functions like synthesis of rRNA
PRKAA1/**N**	Protein Kinase AMP-Activated Catalytic Subunit Alpha 1	Negative regulation of the mTORC1 signalling
**Synaptic transmission**		
KCNJ10/**R**	Potassium Voltage-gated Channel Subfamily J member 10	Regulate neurotransmitter release and Neuronal excitability
KCNH8/**N**	Potassium Voltage-gated Channel Subfamily H	Regulate neurotransmitter release and Neuronal excitability
SNPH/**N**	Syntaphilin	Inhibits SNARE complex formation, mitochondrial trafficking, and neurodegeneration
DLG1/**R**	Discs Large MAGUK Scaffold Protein 1	Modulates synaptic organization during development and Plasticity
PPA2B/LPPR3/**N**	Lipid phosphate phosphatase-related proteins	Putative roles in axonal outgrowth, regeneration, and synaptic plasticity
**Neuronal development and cell-cell interactions**		
NEUROD1/**R**	Neurogenic differentiation 1	Neurogenesis and control of differentiation of neural cell types
EMX1/**N**	Empty Spiracles Homeobox 1	Neurogenesis and control of differentiation of neural cell types
NKX61/**N**	NK6 Homeobox 1	Neurogenesis and control of differentiation of neural cell types
LHX2/**N**	LIM Homeobox 2	Neurogenesis and control of differentiation of neural cell types
NR4A3/**R**	Nuclear Receptor Subfamily 4 Group A Member 3	Mediates CREB-induced neuronal survival
NR2E1/**N**	Nuclear Receptor Subfamily 2 Group E Member 1	Neurogenesis and control of differentiation of neural cell types
ECT2/**N**	Epithelial cell transforming 2	Regulation of neurite outgrowth
BCL6/**N**	B-cell lymphoma 6	Neuroprotection, generation of cortical neurons during embryonic brain development
TIAM1/**N**	T-Cell Lymphoma invasion and metastasis 1	Neuronal migration
NF-kB 2/**R**	Nuclear factor kappa-light-chain-enhancer of activated B cells	Controls transcription of DNA, cytokine production, and cell survival.
BRCA1/**N**	Breast Cancer 1	Positioning of neurons and guides the organization of brain layers to ensure normal development
NCAM 1/**R**	Neural cell adhesion molecule	Role in cell adhesion and synaptic plasticity
DNER/**N**	Delta/Notch Like EGF Repeat Containing	Activation of the NOTCH1 pathway
NLGN1/**N**	Neuroligin 1	Role in synapse function and synaptic signal transmission, and probably mediates its effects by recruiting and clustering other synaptic proteins
UNC5B/**N**	Unc-5 Netrin Receptor B	Involved in pro- and anti-apoptotic processes

^a^Abbreviations: R, genes reported in earlier studies as well as in this study; **N**, novel genes found in this study.

## Discussion

In this study we have identified DNA methylation-regulated potential gene networks, pathophysiological pathways including PDGFR, EGFR, NTRK3 (RTKs), and mTOR signalling in addition to several other potential epilepsy-related genes associated with FCD type II pathology.

Three RTKs, EGFR, PDGFRA, and NTRK3 that govern neuronal and glial development were found to be upregulated in our study. Overexpression of RTKs, TrkB and TrkC has previously been demonstrated in the dysmorphic neurons of FCD^3,5^. Overexpression of EGFR in association with loss of *Tsc1* is reported in mouse as well human TSC brain pathology^24^. Recombinant PDGF-BB has previously been shown to suppress convulsions in an animal model^25^. Downregulation of *NTRK3*, which signals by MAPK pathway upon neurotrophin-3 (NT-3) binding, suggests an important inhibitory role of NT-3 in seizure development and seizure-related synaptic reorganization^25^. As NT-3 acts by binding to NTRK3, downregulation of *NTRK3* observed in our study may have similar effects in FCD. *NTRK3* has also been proposed to be a possible candidate gene in autosomal dominant nocturnal frontallobe epilepsy (ADNFLE)^26^.

Aberrant activation of mTOR pathway is reported in various models of epilepsy, including FCD type II^1^. A range of germline and somatic mutations of PI3K/AKT/mTOR pathway genes leading to mTOR complex 1 (mTORC1) hyperactivation were identified in FCDs^12–16,27,28^. Polyhydramnios, megalencephaly and symptomatic epilepsy (PMSE) has shown to be having a deletion in *STRADα* gene, which regulates AMPK, an energy-sensor protein kinase that controls mTORC1 and TSC2^29^. *PRKAA1*, the catalytic subunit of *AMPK* was downregulated in our study, suggesting inhibition of negative regulation of the mTORC1 complex leading to increased mTORC1 signalling in FCD type II. Similarly, another mTOR signalling molecule *RPS6KA3*, a ribosomal S6 kinase which phosphorylates various substrates, including members of MAPK pathway was found to be upregulated^11^. RPS6KA3 can activate mTOR signalling by directly phosphorylating and therefore inhibiting TSC2 activity to suppress mTOR signalling, or by phosphorylating RPTOR, which regulates mTORC1 activity independent of the PI3K/AKT pathway^12^. It can also phosphorylate RPS6 in response to EGFR signalling which is also modulated in our study, via an mTOR-independent mechanism and may promote translation initiation of proteins involved in synaptic plasticity^11^. Recent studies have shown that phosphorylated RPS6 (p-RPS6), initially believed to be a hallmark of mTOR hyperactivity in FCD type II and TSC pathologies, is actually a marker of dysmorphic and immature neurons in a wide range of pathologies associated with DREs^30–32^. Thus, our current data coupled with several other studies demonstrating the effectiveness of mTOR inhibitors in preventing or reducing seizures in different models of epilepsy suggest possibility of mTOR pathway as a promising therapeutic target for FCD^1,11^.

Notably, two voltage-gated potassium (Kv) channel subunits *KCNH8* and *KCNJ10* were upregulated in our study. Modulation of these Kv channel subunits may play a role in hyperexcitability associated with FCD type II by altering the regulation of the resting membrane as well as action potentials^3,7^. Previous studies have shown modulation of various potassium channels in both animal models and patients with epilepsy^6,7^. Role of *KCNJ10* variants in the pathogenesis of some rare epileptic syndromes is reported^33^. Syntaphilin (*SNPH*) that inhibits SNARE complex formation was found to be downregulated, which may contribute to increased synaptic transmission^34^. A previously proposed biomarker for FCD, *DLG1/SAP97/MAGUK*, was found to be upregulated in this study. High seizure frequency in FCD IIB patients might increase *SAP97* expression thereby influencing both synaptic input and neuronal architecture leading to the morphologic changes^35^. *PPAP2B* (*LPPR3*), a member of plasticity-related genes that have been predicted to participate in axonal outgrowth, regeneration and synaptic plasticity, was found to be upregulated in this study. LPPR4 (PRG1) may play a potential role in epileptogenesis by modulating presynaptic LPA2 receptor signalling-dependent hippocampal excitability^7^.

Methylation-dependent differential expression of several transcription factors, mostly those controlling generation and identity of different cell types in the nervous system during development, was observed in this study. Homeobox gene *EMX1*, a marker of the pyramidal cell lineage, and *LHX2* and *NEUROD1* – two genes involved in neurogenesis were downregulated. *NEUROD1* has previously been shown to be associated with aberrant hippocampal neurogenesis in animal models of epilepsy^6^. *NKX6-1* gene, which controls the generation and identity of different types of cortical GABAergic interneurons, was upregulated. Modulation of *NKX6-1* might be associated with altered spatio-temporal developmental profile of cortical GABAergic interneurons in FCD type II^36^. Two nuclear receptors *NR4A3* and *NR2E1* were downregulated. Importantly, *NR4A3* could be a potential candidate molecule since it mediates CREB-induced neuronal survival and previous studies have suggested role of *NR4A3* (*nor-1*) in regulating hippocampal axon guidance, pyramidal cell survival, and seizure susceptibility^37^. *NR2E1* plays important role in neurogenesis and deletion of the *NR2E1* impairs synaptic plasticity and dendritic structure in the mouse dentate gyrus^38^. Two guanine nucleotide exchange factors – *TIAM1*, which is involved in neuronal migration and ECT2, which plays role in the regulation of neurite outgrowth – were downregulated and upregulated, respectively^39,40^. *BCL6*, a potential CREB target gene and a key factor in the generation of cortical neurons during embryonic brain development was found to be upregulated in our study, hinting at the possibility of its involvement in neuroprotective pathways^41^. We found upregulation of two nuclear factors *NF-kB2* and *BRCA1*. *NFkB* has been previously reported to be upregulated in TLE (HS) patients^6^. Multiple signal transduction events, initiated by inflammation, immunity, differentiation, cell growth, tumorigenesis, and apoptosis, converge on NFkB activation^6^. Thus NFkB could have a potential role in the pathophysiology of FCD type II patients. Recently *BRCA1* gene was shown to play role in the positioning of neurons and guiding the organization of brain layers during development^42^. Also, *BRCA1* mutations in some breast cancer patients were proposed to be associated with seizures^42^. The neural cell adhesion molecule (*NCAM-1*) previously reported as a potential biomarker for drug-effective epilepsy and DRE was also upregulated in this study^43^. *UNC5B*, a netrin receptor with crucial rolein cortical interneuron guidance was upregulated. Since defects in cortical interneuron migration are shown to cause seizures in mice, *UNC5B* could be a potential target in FCD^44^. *DNER*, an activator of the NOTCH1 pathway was upregulated and can play a potential role by mediating neuron-glia interaction during astrocytogenesis^45^. *NLGN1*, (Neuroligin 1) a cell surface protein involved in cell-cell-interactions via its interactions with neurexin family members was also upregulated. Functionally, *NLGN1* overexpression has been shown to enhance excitatory but not inhibitory synaptic transmission^46^. We propose that these epigenetically modulated genes involved in neuronal development and cell-cell interactions might have significant clinical relevance and therapeutic importance in FCD type II.

Conclusively, this study indicates activation of RTKs (*EGFR, PDGFRA, NTRK3*), mTOR (*RPS6KA3, PRKAA1*) signalling, and numerous other potential candidate genes involved in neuronal survival and neuronal migration in FCD type II patients. Our previous RNAseq-based studies revealed major hubs in MTLE-HS patients, supporting the intrinsic severity hypothesis of drug resistance^7^. This study suggests that these epigenetically modified pathways may also account for the drug resistance mechanisms by increasing the severity/chronicity of the disease. Prospective studies based on immunohistochemistry will help to decipher the exact epigenetically modulated gene level changes in specific cell populations. Further, we have found upregulation of *DNMT3α* in these patients. It is known that DNMTs can modulate synaptic plasticity in hippocampus^20^. Our result suggests that *DNMT3α* might modulate synaptic plasticity by epigenetically regulating EGFR, PDGFRA, mTOR pathways, in addition to other genes involved in cortical development and neuronal plasticity, and thus could play a critical pathogenetic role in epileptogenesis in FCD type II patients. Efficacy of ketogenic diet is reported in a range of childhood-onset epilepsies including FCD. Recent experimental data also suggest that the ketogenic diet attenuates DNA methylation^47^. Thus DNA methylation regulators (DNMT3α) and/or DNA methylation modulated genes such as RTKs and mTOR pathway molecules could be possible anti-epileptogenic targets.

In summary, this study identifies several candidate genes with a potential role as diagnostic/prognostic biomarkers and may help tailor the treatment approach for an effective seizure control in patients suffering from DRE caused by FCD type II pathology. The current study has few limitations. We have validated our MeDIP and RNAseq data on a larger cohort but the sample size for MeDIP array and RNAseq was limited. A small sample size also prevents us from a type-specific (FCD type IIa vs type IIb) analysis and drawing any useful conclusions. Also, we have only discussed the CpG islands in the upstream promoter regions, as the role of other CpG islands in epigenetic regulation of gene expression is not well-defined. For example, altered DNA methylation of gene bodies has previously been reported, including in rat models of epilepsy but with no clear correlation with respect to its effect on gene expression changes^18,48^. Another limitation of this study was the unavailability of age- and region-matched non-epileptic control samples. Future experimentations addressing these limitations are likely to provide us with a better knowledge of the underlying mechanisms and pharmacological targets of FCD type II pathologies.

## Materials and Methods

### Patients

The patients who were diagnosed to have DRE due to FCD type II pathology with more extensive changes in the frontal lobes and underwent electrocorticography (ECoG)-guided surgery were included in the study **(**Table 3**)**. Patient’s age at surgery ranged from 5 to 38 years (mean age 18.5 ± 9.6 years). The pathology in each patient was confirmed by magnetic resonance imaging (MRI), fluoro-2-deoxyglucose positron emission tomography (FDG-PET), and ECoG evaluations. Patients with dual pathology were excluded. The resection was based on clinical evaluations and pathology was further verified by histopathologic examinations as described previously (Supplementary Fig. S2)^49^.

**Table 3:** Clinical data of controls and FCD type II patients.

Patient/Control^a^	Age (years)	Sex	Pathology/COD^b^	Antiepileptic drugs^c^
A1	25	M	Pelvic injury	NA
A2	18	F	Pelvic injury and lower limb injury	NA
A3	16	M	Abdominal injury	NA
A4	18	M	Head and abdominal injury	NA
A5	40	M	Respiratory failure	NA
A6	36	F	Heart failure	NA
A7	26	F	Pulmonary carcinoma	NA
A8	19	F	Renal carcinoma	NA
A9	33	M	Abdominal injury	NA
A10	8	M	Heart failure	NA
F1	13	F	FCD typeIIb	CLO, PHT, LEV
F2	18	M	FCD typeIIa	CBZ, LEV, TPR
F3	24	F	FCD typeIIb	CLN, VPA, LEV, VBN, ZNS
F4	6	M	FCD typeIIa	VPA, CLO
F5	5	F	FCD typeIIa	CBZ, CLO, LEV
F6	13	M	FCD typeIIa	CLO, CLN, LTG, LEV, VPA
F7	24	F	FCD typeIIb	LTG, VPA, TPR
F8	16	F	FCD typeIIb	VPA, LEV
F9	28	M	FCD typeIIb	CBZ, CLO
F10	38	M	FCD typeIIa	CLO, PHT, LEV

^a,b,c^Abbreviations: A, Autopsy control; F, FCD; COD, Cause of death; CBZ, Carbamazepine; CLO, Clobazam; CLN, Clonazepam; LEV, Levetiracetam; LTG, Lamotrigine; PHT, Phenytoin; TPR, Topiramate; VPA, Valproinic acid; ZNS, Zonisamide; VBN, Vigabatrin; Not Applicable, NA.

### Controls

As there are no “ideal” or acceptable non-epilepsy controls for such studies involving epilepsy, we have used histologically normal cortex tissues (data not shown) obtained from the frontal lobes of the post-mortem cases without any history of seizures or other neurological disorders as non-epileptic controls **(**Table 2**)**. Autopsy patient’s age ranged from 8 to 40 years (mean age 23.9 ± 9.5 years). All the autopsies were performed within 8 h of death. The tissues obtained within this post-mortem interval are documented to yield DNA and RNA suitable for genome-wide studies^50^.

### Tissue preparation

Resected brain tissues were immediately divided into two parts: one portion was immediately snap-frozen and stored at −70 °C until further processing and the second portion was fixed in 10% buffered formalin for histopathological examinations.

Tissue samples taken from 3 FCD patients (F1-F3) and 2 autopsy controls (A1-A2) were processed for DNA methylation and RNAseq analysis. An independent set of 7 patient (F4-F10) and 8 controls (A3-A10) along with (F1-F3) and (A1-A2) tissue samples was used for validation of DNA methylation as well as RNAseq data by qPCR analysis.

### Genome-wide DNA methylation analysis

Genomic DNA was extracted from the frozen tissues using QIAAmp DNA Mini kit (Qiagen, Hilden, Germany) following manufacturer’s protocol and methylated DNA was immunoprecipitated as described previously with some modifications^21^. Briefly, for methylated CpG island microarrays, 6 μg of genomic DNA was digested with *Mse*I restriction enzyme (New England Biolabs, MA, USA) and immunoprecipitated with 10 µg of monoclonal mouse anti-5-methylcytidine antibody (Abcam, Cambridge, UK) or a negative control normal mouse IgG (Abcam, #ab81032). The immunoprecipitated DNA and a sample of input DNA were amplified using GenomePlex Complete Whole Genome Amplification kit (Sigma-Aldrich, MO, USA). Subsequently, 2 μg of DNA from the WGA amplified sample (MeDIP-WGA), along with their corresponding whole genomic DNA (Input), were labeled using the SureTag DNA labeling kit (Agilent #5190-3400) and hybridized to Agilent Human DNA Methylation Microarray slides (G4495A, AMAIDID 023795) containing 237,227 biological probes covering all annotated 27,627 CpG islands and 5081 UMR regions with median probe spacing of 97 bp, following the manufacturer’s instructions. The slides were subsequently washed and scanned using Agilent scanner P/N G2565BA and each 5-μm image extracted using Agilent Features Extraction software (version 10.0) and methylation status analysis was carried out using GeneSpring GX software (version 13.0). The signal values were transformed to the log2, and then quantile and percentile shifts were applied to obtain equal distributions of the probe signal intensities and median-normalized log2 enrichment ratios (MeDIP/Input) were calculated. Differentially methylated genes between the control and FCD groups were identified using an adjusted Student’s *t*-test (*p* < 0.05) corrected for multiple comparisons with the false discovery rate (FDR) and fold-change (FC) cut off of ≥2.

### RNAseq methodology and analysis

RNAseq analysis was carried out on Illumina platform as described previously^7^. Briefly, brain samples were homogenized in Trizol and total RNA was isolated from cells using RNeasy Kit (Qiagen, Hilden, Germany) according to the manufacturer’s protocol. An additional *DNase*1 digestion step was performed to ensure that the samples were not contaminated with genomic DNA. RNA purity was checked by calculating RIN in Agilent BioAnalyzer 2100 (Agilent, CA, USA). Transcriptome sequencing libraries were prepared using the TruSeq RNA Access Library Prep Kit (Illumina, CA, USA). Paired-end sequencing (100 bp) was performed at an Illumina HiSeq 2500 instrument. All the 100 bp paired-end raw reads were quality checked for low- quality bases and adapter sequences. Quality check was done using NGS QC Toolkit (version 2.3.1)^51^. Low- quality bases and reads were filtered out from the datasets prior to read mapping. The paired-end reads were aligned to the reference human genome, Feb. 2009 release, downloaded from UCSC database (GRCh37/hg19). Alignment was performed using HISAT (a fast spliced aligner with low memory requirements) program (version 0.1.7). The aligned reads were used for estimating expression of genes and transcripts using cufflinks program (version 2.2.1) and are reported as FPKM/RPKM (Fragment per kilo per million/Read per kilo per million) units for each of the genes and transcripts. Differential gene expression analysis was performed using cuffdiff (version 2.2.1). Significant mRNA fold-change was determined by an adjusted *p*-value lower than 0.05 based on the Benjamini and Hochberg multiple testing correction.

### Integrative analysis of DNA methylation and gene expression

To identify those DNA methylation changes associated with concomitant changes in gene expression, integrative analysis of DNA methylation and RNAseq data was carried out using GeneSpring GX software (version 13.0). Fold-change for DGE analysis was recalculated using GeneSpring GX software (version 13.0). Absolute FPKM values from RNAseq data for each FCD patient and control samples were used for the comparative analysis between the control and FCD group. The gene expression data was log2 transformed and normalized using quantile normalization. Probes with an adjusted *p*-value less than 0.05 and log2FC of 1 were selected. The correlation of log2enrichment ratio values obtained from the DNA methylation arrays (*p* < 0.05, log2FC ≥ 1) and the fold-change in expression (*p* < 0.05, log2FC ≥ 1) of the identified genes was used as an indicator of the correlation between DNA methylation and gene expression. The probes showing hypomethylation (log2FC ≤ (−)1, *p* < 0.05) in conjunction with higher expression (log2FC > 1, *p* < 0.05) and the probes exhibiting hypermethylation (log2FC  ≥ 1, *p* < 0.05) in conjunction with lower expression (log2FC < (−)1, *p* < 0.05) were also identified.

### Functional classification/gene network analyses

Set of genes with concordant DNA methylation and expression patterns were analyzed for gene Ontology (GO) enrichments using the DAVID (version 6.7) tool to cluster differentially-regulated genes by their common functionality. Enriched ontologies were identified and statistically clustered by comparison of their constituent genes. Clustering enrichment thresholds required DAVIDEASE scores such that the modified Fischer Exact *p*-value was less than 0.05^52^. Pathway analysis was carried out with MetaCore software (GeneGo Inc, Michigan, USA) with a FDR adjustment to adjust the *p-*value. Based on the GO enrichment scores and pathway analysis, 25 genes were filtered for further network analysis using natural language processing-based (NLP) network discovery algorithms in GeneSpring GX software (version 13.0) as described in GeneSpring GX 10 manual^53^. The software overlaid the list of significantly altered 25 genes onto a global molecular network developed from information contained in the knowledge base to derive networks in which the focus genes are projected as nodes and interacting partners are clustered around these nodes based on their reported connectivity.

### *DNMT1* and *DNMT3a* expression and validation of DNA methylation and RNAseq data

Quantitative real-time PCR (qPCR) was performed to evaluate the expression of *DNMT1* and *DNMT3α* and validate the expression of differentially methylated and differentially expressed genes using specific primers for selected genes on 10 FCD patients and 10 control samples. Specific primers for selected genes **(**Supplementary Table S4**)** were designed using NCBI Primer-BLAST (version 0.4.0). For MeDIP validation, previously published primers for *GAPDH* unmethylated and *H19* imprinted were used as negative and positive controls^54^. All samples were amplified in triplicates. Purified RNA (as described above) was reverse-transcribed using High Capacity cDNA Reverse Transcription kit (Invitrogen, CA, USA) following the manufacturer’s protocol. For validation of DNA methylation analysis, DNA was extracted and MeDIP was carried out as described above. Both input and MeDIP-DNA were used as templates in the qPCR reactions. A negative control of MeDIP-DNA using normal mouse IgG (Santa Cruz Biotechnology, #SC-2025) was also included. In order to normalize qPCR reactions for gene expression analysis, *HPRT* (hypoxanthine phosphoribosyl-transferase) was included as reference gene^55^. Real-time PCR amplifications were performed in CFX96 real-time systems (Bio-Rad, CA, USA) with the following cycling parameters: an initial hot start of 95 °C for 3 min followed by 40 cycles of 95 °C for 5 s and 60 °C for 30 s. The 2^−ΔΔCq^ method was used to quantify the relative normalized expression of studied genes based on the average Ct values across samples^56^. Test of significance was performed using Student’s t-test (two-tailed, unpaired) and *p*-values < 0.05 were considered significant.

### Ethical Statement

We confirm that we have read the Journal’s position on issues involved in ethical publication and affirm that this report is consistent with those guidelines. This study was reviewed and approved by Institutional Ethics Committee (IEC), All India Institute of Medical Sciences (AIIMS), New Delhi, India, and Institutional Human Ethics (IHE), National Brain Research Centre, Manesar, India, before the study began. Based on the concordant observations, decision for surgical resection was taken after explaining the available options and obtaining the informed written consent from the patient or their legal guardians.

## Electronic supplementary material

Supplementary Information

## Data Availability

RNAseq data and sequences are submitted at NCBI BioProject (http://www.ncbi.nlm.nih.gov/bioproject/PRJNA369732) and DNA methylation data have been deposited in NCBI Gene Expression Omnibus and are accessible through GEO Series accession number GSE96067.
