# Supplementary material for: Genome-wide DNA Methylation and RNAseq Analyses Identify Aberrant Signalling Pathways in Focal Cortical Dysplasia (FCD) Type II
Source: Sci Rep. 2018 Dec 19;8:17976. doi: 10.1038/s41598-018-35892-5 (PMC6299275; doi:10.1038/s41598-018-35892-5)
Supplement: Supplementary file 1 — Supplementary Information [file 41598_2018_35892_MOESM1_ESM.pdf]

Genome-wide DNA Methylation and RNAseq Analyses Identify Aberrant Signalling Pathways in Focal Cortical Dysplasia (FCD) Type II

Aparna Banerjee Dixit[1,2*], Devina Sharma[1,4], Manjari Tripathi[1,3], Arpna Srivastava[4], Debasmita Paul[1,4], Deepak Prakash[5], Chitra Sarkar[6], Krishan Kumar[1,4], Jyotirmoy Banerjee[1,7], and P Sarat Chandra[1,4**]

1. Center of Excellence for Epilepsy, A joint NBRC-AIIMS collaboration, NBRC, Manesar, India
2. Dr. B R Ambedkar Center for Biomedical Research, University of Delhi, Delhi, India
3. Department of Neurology, AIIMS, New Delhi, India
4. Department of Neurosurgery, AIIMS, New Delhi, India
5. Department of Forensic Medicine and Toxicology, AIIMS, New Delhi, India
6. Department of Pathology, AIIMS, New Delhi, India
7. Department of Biophysics, AIIMS, New Delhi, India

[**]**Corresponding Author:** P. Sarat Chandra, Room No 605, Department of Neurosurgery, AIIMS, New Delhi, India-110029, Tel.: +91-11-26546477, Fax: +91-11-26588248.
Email: saratpchandra3@gmail.com

[*]**Co-Corresponding Author:** aparnabanerjeedixit@gmail.com

Table S1. DNA methylation summary showing number of genes with altered methylation in CpG sites in FCD cases as compared to controls.

	**All CpG sites (*p* <0.05 & FC ≥2)**	**CpG sites in promoter region**	**CpG sites in promoter and concordant with gene expression**
**Hypermethylated**	12,686	3,656	104
**Hypomethylated**	6,402	2,069	72
**Total**	19,088	5,725	176

Table S2. RNAseq summary.

Sample ID	Total raw reads	Total processed reads	Total aligned reads (Homo sapiens genome)	Differentially expressed genes (C vs FCD) (q <0.05 and FC ≥2)	
				Upregulated	Downregulated
A1	56,355,740	44,840,404	42,544,575		
A2	64,403,632	57,897,550	52,165,758		
F1	34,863,534	24,796,108	22,663,924	386	220
F2	37,629,246	29,382,142	24,883,116		
F3	31,892,308	23,717,680	22,624,249		

Table S3. List of DEGs with concordant promoter methylation patterns in FCD type II patients.

Gene ID	Ensembel Transcript ID	Gene Name	Log2FC Methylation	Log2FC RNA Seq
SLC24A2	ENSG00000155886	Solute Carrier Family 24 Member 2	-4.78583	1.30394
AGTPBP1	ENSG00000135049	ATP/GTP Binding Protein 1	-4.56773	2.21165
MTM1	ENSG00000171100	Myotubularin 1	-4.35996	1.28522
DNER	ENSG00000187957	Delta/Notch Like EGF Repeat Containing	-3.9897	1.16415
MTX3	ENSG00000177034	Metaxin 3	-3.96591	1.88101
DDR1	ENSG00000204580	Discoidin Domain Receptor Tyrosine Kinase 1	-3.86534	1.54253
WDR91	ENSG00000105875	WD Repeat Domain 91	-3.73377	1.78979
MANEA	ENSG00000172469	Mannosidase Endo-alpha	-3.63457	2.388
EGFR	ENSG00000146648	Epidermal Growth Factor Receptor	-3.56741	1.04186
ITGB8	ENSG00000105855	Integrin Subunit Beta 8	-3.45843	1.32933
RPS6KA3	ENSG00000177189	Ribosomal Protein S6 Kinase A3	-3.42965	1.22167
PDGFRA	ENSG00000134853	Platelet Derived Growth Factor Receptor Alpha	-3.36653	1.97296
PPAP2B	ENSG00000162407	Phosphatidic Acid Phosphatase Type 2B	-3.36087	1.19268
ADIPOR2	ENSG00000006831	Adiponectin receptor 2	-3.33256	1.53665
GALNT7	ENSG00000109586	Polypeptide N-Acetylgalactosaminyltransferase 7	-3.28518	2.01707

CNTN2	ENSG00000184144	Contactin 2	-3.16096	2.45969
ABCC9	ENSG00000069431	ATP Binding Cassette Subfamily C Member 9	-3.15363	1.63625
UNC5B	ENSG00000107731	Unc-5 Netrin Receptor B	-3.13645	1.9954
PAPSS1	ENSG00000138801	3'-Phosphoadenosine 5'-Phosphosulfate Synthase 1	-3.12888	0.964837
DDX6	ENSG00000110367	DEAD-Box Helicase 6	-3.10188	1.54029
LUZP2	ENSG00000187398	Leucine Zipper Protein 2	-3.07717	1.39464
LAMP1	ENSG00000185896	Lysosomal Associated Membrane Protein 1	-3.03868	1.31406
NCAM1	ENSG00000149294	Neural Cell Adhesion Molecule 1	-3.02435	1.1772
PCDH10	ENSG00000138650	Protocadherin 10	-2.96505	1.15417
ADAMTS1	ENSG00000140873	ADAM Metallopeptidase With Thrombospondin Type 1 Motif 1	-2.94269	4.58769
PIGK	ENSG00000142892	Phosphatidylinositol Glycan Anchor Biosynthesis Class K	-2.93308	2.61827
ENPP5	ENSG00000112796	EctonucleotidePyrophosphatase/Phosphodiesterase 5	-2.89793	1.31192
NPC1	ENSG00000141458	NPC Intracellular Cholesterol Transporter 1	-2.89198	2.46624
KCNJ10	ENSG00000177807	Potassium Voltage-Gated Channel Subfamily J Member 10	-2.83805	1.18424
GOLGA7	ENSG00000147533	Golgin A7	-2.8341	1.62645
PTPLAD1	ENSG00000074696	3-Hydroxyacyl-CoA Dehydratase 3	-2.81722	1.22123
ARRDC2	ENSG00000105643	Arrestin Domain Containing 2	-2.77383	1.55281

IREB2	ENSG00000136381	Iron Responsive Element Binding Protein 2	-2.76994	1.20001
SEMA6A	ENSG00000092421	Semaphorin 6A	-2.76171	2.38162
LIPA	ENSG00000107798	Lipase A, Lysosomal Acid Type	-2.74931	3.01443
TP53INP2	ENSG00000078804	Tumor Protein P53 Inducible Nuclear Protein 2	-2.72875	1.42095
MMGT1	ENSG00000169446	Membrane Magnesium Transporter 1	-2.7156	1.22319
PPA1	ENSG00000180817	Pyrophosphatase (Inorganic) 1	-2.65418	1.22739
ITGB1	ENSG00000150093	Integrin Subunit Beta 1	-2.62181	2.34886
GLG1	ENSG00000090863	Golgi Glycoprotein 1	-2.62104	0.952517
NKD1	ENSG00000140807	Naked Cuticle Homolog 1	-2.60755	2.0399
ICOSLG	ENSG00000160223	Inducible T-Cell Costimulator Ligand	-2.55181	3.23343
ECT2	ENSG00000114346	Epithelial Cell Transforming 2	-2.5158	1.69768
NKX6-2	ENSG00000148826	NK6 Homeobox 2	-2.49747	2.23546
PDZD8	ENSG00000165650	PDZ Domain Containing 8	-2.48744	1.05886
LBR	ENSG00000143815	Lamin B Receptor	-2.46761	1.57228
PRKAA1	ENSG00000132356	Protein Kinase AMP-Activated Catalytic Subunit Alpha 1	-2.45817	1.50616
ARHGAP20	ENSG00000137727	Rho GTPase Activating Protein 20	-2.45771	1.75825
SGMS1	ENSG00000198964	Sphingomyelin Synthase 1	-2.42887	2.19562

CREB5	ENSG00000146592	CAMP Responsive Element Binding Protein 5	-2.41802	2.67727
RAP1B	ENSG00000127314	RAP1B, Member Of RAS Oncogene Family	-2.41717	1.44292
VHL	ENSG00000134086	Von Hippel-Lindau Tumor Suppressor	-2.4155	1.39591
RASSF2	ENSG00000101265	Ras Association Domain Family Member 2	-2.39945	1.83446
NDE1	ENSG00000072864	NudE Neurodevelopment Protein 1	-2.3938	1.5911
TCP11L2	ENSG00000166046	T-Complex 11 Like 2	-2.39358	3.37428
HCFC2	ENSG00000111727	Host Cell Factor C2	-2.3926	1.54538
CBFB	ENSG00000067955	Core-Binding Factor Beta Subunit	-2.3746	1.45086
BRCA1	ENSG00000012048	BRCA1, DNA Repair Associated	-2.36799	1.07227
CYB5R2	ENSG00000166394	Cytochrome B5 Reductase 2	-2.36623	3.26725
TMED10	ENSG00000170348	Transmembrane P24 Trafficking Protein 10	-2.36496	1.9869
DLG1	ENSG00000075711	Discs Large MAGUK Scaffold Protein 1	-2.35808	1.1261
EDIL3	ENSG00000164176	EGF Like Repeats And Discoidin Domains 3	-2.35307	3.62261
SCIN	ENSG00000006747	Scinderin	-2.35064	2.1598
SYT15	ENSG00000204176	Synaptotagmin 15	-2.33849	1.68693
PSEN1	ENSG00000080815	Presenilin 1	-2.32846	1.78596

ERMP1	ENSG00000099219	Endoplasmic Reticulum Metallopeptidase 1	-2.32258	1.7304
FAM105A	ENSG00000145569	Family With Sequence Similarity 105 Member A	-2.31272	2.0601
ATP10A	ENSG00000206190	ATPase Phospholipid Transporting 10A	-2.31182	1.71843
NALCN	ENSG00000102452	Sodium Leak Channel, Non-Selective	-2.30864	1.09973
NLGN1	ENSG00000169760	Neuroligin 1	-2.30045	1.23066
TM9SF3	ENSG00000077147	Transmembrane 9 Superfamily Member 3	-2.29048	1.28625
KCTD12	ENSG00000178695	Potassium Channel Tetramerization Domain Containing 12	-2.28774	2.10302
KCNH8	ENSG00000183960	Potassium Voltage-Gated Channel Subfamily H Member 8	-2.28019	4.19224
CLN8	ENSG00000182372	Ceroid-Lipofuscinosis, Neuronal 8	-2.2756	1.54466
MATN3	ENSG00000132031	Matrilin 3	-2.27018	2.45811
ACSL4	ENSG00000068366	Acyl-CoA Synthetase Long-Chain Family Member 4	-2.26902	1.12845
GDI2	ENSG00000057608	GDP Dissociation Inhibitor 2	-2.25938	0.946978
AGAP1	ENSG00000157985	ArfGAP With GTPase Domain, Ankyrin Repeat And PH Domain 1	-2.25928	1.18361
ZNF669	ENSG00000188295	Zinc Finger Protein 669	-2.25597	2.56273
SRBD1	ENSG00000068784	S1 RNA Binding Domain 1	-2.24817	1.677
C1GALT1C1	ENSG00000171155	C1GALT1 Specific Chaperone 1	-2.239	1.85808

UTRN	ENSG00000152818	Utrophin	-2.23042	1.65485
SMCHD1	ENSG00000101596	Structural Maintenance Of Chromosomes Flexible Hinge Domain Containing 1	-2.22994	1.47409
ABTB2	ENSG00000166016	Ankyrin Repeat And BTB Domain Containing 2	-2.19933	1.48483
USO1	ENSG00000138768	USO1 Vesicle Transport Factor	-2.19856	1.05194
GNB4	ENSG00000114450	G Protein Subunit Beta 4	-2.19843	1.73648
LITAF	ENSG00000189067	Lipopolysaccharide Induced TNF Factor	-2.1935	2.05305
NEO1	ENSG00000067141	Neogenin 1	-2.15867	1.15277
C9orf3	ENSG00000148120	Chromosome 9 Open Reading Frame 3	-2.15784	1.30292
SALL1	ENSG00000103449	Spalt Like Transcription Factor 1	-2.15531	1.40662
SKAP2	ENSG00000005020	Src Kinase Associated Phosphoprotein 2	-2.15335	1.44454
EMILIN2	ENSG00000132205	Elastin MicrofibrilInterfacer 2	-2.15037	2.20526
MICALL1	ENSG00000100139	MICAL Like 1	-2.1265	2.21593
GAB2	ENSG00000033327	GRB2 Associated Binding Protein 2	-2.11879	1.50588
PAIP2B	ENSG00000124374	Poly(A) Binding Protein Interacting Protein 2B	-2.11586	1.41248
FMNL2	ENSG00000157827	Formin Like 2	-2.10986	1.31054

H6PD	ENSG00000049239	Hexose-6-Phosphate Dehydrogenase/Glucose 1-Dehydrogenase	-2.08927	1.4464
STT3B	ENSG00000163527	STT3B, Catalytic Subunit Of The Oligosaccharyltransferase Complex	-2.08711	1.30092
OTUD6B	ENSG00000155100	OTU Domain Containing 6B	-2.0839	1.50958
SRP54	ENSG00000100883	Signal Recognition Particle 54	-2.04497	1.24747
IKZF1	ENSG00000185811	IKAROS Family Zinc Finger 1	-2.03404	2.17222
KDM6A	ENSG00000147050	Lysine Demethylase 6A	-2.01854	2.069
RGS10	ENSG00000148908	Regulator Of G-Protein Signaling 10	-2.0111	1.60061
SNX18	ENSG00000178996	Sorting Nexin 18	-1.96698	1.20311
GPR123	ENSG00000197177	Adhesion G Protein-Coupled Receptor A1	5.40033	-1.67675
GALNT9	ENSG00000182870	Polypeptide N-Acetylgalactosaminyltransferase 9	4.804522	-1.52505
TOX2	ENSG00000124191	TOX High Mobility Group Box Family Member 2	4.411525	-1.89784
ARID4A	ENSG00000032219	AT-Rich Interaction Domain 4A	4.351728	-1.07586
TYK2	ENSG00000105397	Tyrosine Kinase 2	4.114358	-2.19267
DGKA	ENSG00000065357	Diacylglycerol Kinase Alpha	3.8528	-2.66016
STK36	ENSG00000163482	Serine/Threonine Kinase 36	3.653658	-2.28527
CHRM1	ENSG00000168539	Cholinergic Receptor Muscarinic 1	3.618404	-1.20513

GLS2	ENSG00000135423	Glutaminase 2	3.611736	-1.94075
C7orf60	ENSG00000164603	C7orf60	3.550055	-1.09372
HERC2P2	ENSG00000140181	Hect Domain And RLD 2 Pseudogene 2	3.497333	-1.35604
STEAP2	ENSG00000157214	STEAP2 Metalloreductase	3.360376	-2.65026
CACNB3	ENSG00000167535	Calcium Voltage-Gated Channel Auxiliary Subunit Beta 3	3.252739	-2.07167
AGPAT2	ENSG00000169692	1-Acylglycerol-3-Phosphate O-Acyltransferase 2	3.22188	-1.7042
SOX9	ENSG00000125398	SRY-Box 9	3.218154	-1.18443
GPR19	ENSG00000183150	G Protein-Coupled Receptor 19	3.187354	-1.49059
NRXN3	ENSG00000021645	Neurexin 3	3.179625	-1.58908
KCNIP2	ENSG00000120049	Potassium Voltage-Gated Channel Interacting Protein 2	3.151674	-1.7704
NOP2	ENSG00000111641	NOP2 Nucleolar Protein	3.050214	-2.04852
RPAP1	ENSG00000103932	RNA Polymerase II Associated Protein 1	3.038714	-1.22722
PPP2R3B	ENSG00000167393	Protein Phosphatase 2 Regulatory Subunit B''Beta	3.001196	-1.22546
BCL6	ENSG00000113916	B-Cell CLL/Lymphoma 6	2.998252	-1.11706
NR2E1	ENSG00000112333	Nuclear Receptor Subfamily 2 Group E Member 1	2.939855	-1.2758
NEUROD1	ENSG00000162992	Neuronal Differentiation 1	2.874308	-2.94055

WDR66	ENSG00000158023	WD Repeat Domain 66	2.850906	-2.69102
GLIS1	ENSG00000174332	GLIS Family Zinc Finger 1	2.831325	-2.7413
FARP1	ENSG00000152767	FERM, ARH/RhoGEF And Pleckstrin Domain Protein 1	2.830282	-2.04798
ZFPM1	ENSG00000179588	Zinc Finger Protein, FOG Family Member 1	2.824425	-1.34212
FAM189A2	ENSG00000135063	Family With Sequence Similarity 189 Member A2	2.811912	-1.28981
FAM110A	ENSG00000125898	Family With Sequence Similarity 110 Member A	2.797008	-1.61545
MGAT5B	ENSG00000167889	Mannosyl (Alpha-1,6-)-Glycoprotein Beta-1,6-N-Acetyl-Glucosaminyltransferase, Isozyme B	2.781685	-1.6051
MSX1	ENSG00000163132	MshHomeobox 1	2.773261	-2.90563
THOC2	ENSG00000125676	THO Complex 2	2.753872	-1.5444
LRFN5	ENSG00000165379	Leucine Rich Repeat And Fibronectin Type III Domain Containing 5	2.716158	-1.2407
TIAM1	ENSG00000156299	T-Cell Lymphoma Invasion And Metastasis 1	2.66889	-1.85802
GLI4	ENSG00000250571	GLI Family Zinc Finger 4	2.659751	-1.79947
RALBP1	ENSG00000017797	RalA Binding Protein 1	2.659246	-2.089
FGFRL1	ENSG00000127418	Fibroblast Growth Factor Receptor-Like 1	2.644448	-1.08006
POU3F1	ENSG00000185668	POU Class 3 Homeobox 1	2.634766	-1.91835

COLEC11	ENSG00000118004	Collectin Subfamily Member 11	2.623136	-2.57383
TESK1	ENSG00000107140	Testis-Specific Kinase 1	2.582726	-1.40071
NFKB2	ENSG00000077150	Nuclear Factor Kappa B Subunit 2	2.581455	-1.89876
DERL3	ENSG00000099958	Derlin 3	2.576996	-1.7985
LRRC6	ENSG00000129295	Leucine Rich Repeat Containing 6	2.55004	-1.73143
ARID5A	ENSG00000196843	AT-Rich Interaction Domain 5A	2.509094	-1.16718
LY6G5C	ENSG00000204428	Lymphocyte Antigen 6 Complex, Locus G5C	2.487686	-1.90609
CLK3	ENSG00000179335	CDC Like Kinase 3	2.466123	-1.2545
NR4A3	ENSG00000119508	Nuclear Receptor Subfamily 4 Group A Member 3	2.456201	-1.77488
TPD52L2	ENSG00000101150	Tumor Protein D52 Like 2	2.441505	-1.23541
H19	ENSG00000130600	H19, Imprinted Maternally Expressed Transcript	2.435496	-3.11917
ST8SIA5	ENSG00000101638	ST8 Alpha-N-Acetyl-Neuraminide Alpha-2,8-Sialyltransferase 5	2.428272	-1.28915
SNPH	ENSG00000101298	Syntaphilin	2.387001	-1.2076
SLC25A39	ENSG00000013306	Solute Carrier Family 25 Member 39	2.372125	-1.67356
ACHE	ENSG00000087085	Acetylcholinesterase	2.361224	-1.83798
LRRC37A3	ENSG00000176809	Leucine Rich Repeat Containing 37 Member A3	2.358471	-1.44329
DDX41	ENSG00000183258	DEAD-Box Helicase 41	2.32344	-1.31096

BEGAIN	ENSG00000183092	Brain Enriched Guanylate Kinase Associated	2.27194	-1.18377
ZNF579	ENSG00000218891	Zinc Finger Protein 579	2.271508	-1.32218
CRABP2	ENSG00000143320	Cellular Retinoic Acid Binding Protein 2	2.229529	-1.58825
NRSN2	ENSG00000125841	Neurensin 2	2.213961	-1.57654
CPLX1	ENSG00000168993	Complexin 1	2.202647	-1.37905
NTRK3	ENSG00000140538	Neurotrophic Receptor Tyrosine Kinase 3	2.201488	-2.22575
PCDH8	ENSG00000136099	Protocadherin 8	2.199782	-1.73291
PCNXL2	ENSG00000135749	Pecanex Homolog 2	2.190312	-2.35161
TRIM33	ENSG00000197323	Tripartite Motif Containing 33	2.177755	-2.51862
TGIF1	ENSG00000177426	TGFB Induced Factor Homeobox 1	2.163086	-1.32136
METRNL	ENSG00000176845	Meteorin Like, Glial Cell Differentiation Regulator	2.157485	-1.22302
EMX1	ENSG00000135638	Empty Spiracles Homeobox 1	2.154409	-2.20843
LRFN1	ENSG00000128011	Leucine Rich Repeat And Fibronectin Type III Domain Containing 1	2.116891	-1.37621
LHX2	ENSG00000106689	LIM Homeobox 2	2.111085	-1.26146
PRR14	ENSG00000156858	Proline Rich 14	2.106508	-1.68079

CORO6	ENSG00000167549	Coronin 6	2.088644	-2.53626

Table S4. Real-time PCR primers.

Gene	Primers (5'-3')	Amplicon (bp)
EGFR	**F-** CAGCGCTACCTTGTCATTCA	195
	**R-** TGCACTCAGAGAGCTCAGGA	
NEUROD1	**F-** GTTCTCAGGACGAGGAGCAC	164
	**R-** CTTGGGCTTTTGATCGTCAT	
RPS6KA3	**F-** GTTGCAGTGAGCCAAGATCA	166
	**R-** AGCTTCTGTGGCAGGAAAAA	
BRCA1	**F-** TAGGGCTGGAAGCACAGAGT	203
	**R-** AATTTCCTCCCCAATGTTCC	
NR4A3	**F-** CCCCTCCAGGTTCCAGTTAT	205
	**R-** ATTTGGTACACGCAGGAAGG	
NTRK3	**F-** ACTTCCGTCAGGGACACAAC	219
	**R-** CCTCCCTCTGGAAATCCTTC	
BCL6	**F-** GATGAGATTGCCCTGCATTT	203
	**R-** TTCTTCCAGTTGCAGGCTTT	
DLG1	**F-** CCAGGCAGGTTACACCAGAT	211
	**R-** TCCTGCTCACTCTGGTCCTT	
PRKAA1	**F-** GGAGCCTTGATGTGGTAGGA	183
	**R-** TTTCATCCAGCCTTCCATTC	
HPRT	**F-** GCTTTCCTTGGTCAGGCAGTA	74
	**R-** GGTCCTTTTCACCAGCAAGCT	
H19	**F-** ATCCCCAGCCTTTTACTGAACT	288
	**R-** CCGAGCCATCGAACATCCTA	
GAPDH	**F-** CCACAGTCCAGTCCTGGGAACC	183
	**R-** GAGCTACGTGCGCCCGTAAAA	
DNMT1	**F-** GTGGGGGACTGTGTCTCTGT	204
	**R-** TGAAAGCTGCATGTCCTCAC	
DNMT3a	**F-** CCGGAACATTGAGGACATCT	162
	**R-** CAGCAGATGGTGCAGTAGGA	

FIGURE LEGENDS

Figure S1: Top ten pathway maps identified by MetaCore in the 177 genes showing inverse correlation with the CpG methylation patterns in the promoter region. The list is arranged per descending *p*-value score.

Figure S2: Histological appearance of representative FCD type IIa and IIb tissues (ILAE classification). FCD type IIA **(a,b)**: Low power magnification of cortex showing disrupted cortical lamination (H&E, x100) **(a)** with presence of dysmorphic neurons with enlarged cell body and nucleus, and abnormal peripheral distribution of Nissl substance (H&E, x200) **(b)**. FCD type IIB **(c,d)**: H&E section of cortex shows severe cortical dyslamination with cytologically abnormal neurons (x100) **(c)**; along with presence of balloon cells (arrows) (H&E, x200) **(d)**.

Figure S3: Graphical display of the various type of associations between different epileptic genes. (A) Different colored edges with arrows show the direction of the interactions and circles with + and – symbols represent positive and negative regulation. **(B)** Various entities not reported in our study but are associated with the epileptic genes (56) are enclosed in different colored shapes. **(C)** Various edges and symbols (small squares, triangles and circles) shows diiferent modes of regulations.

Figure S1

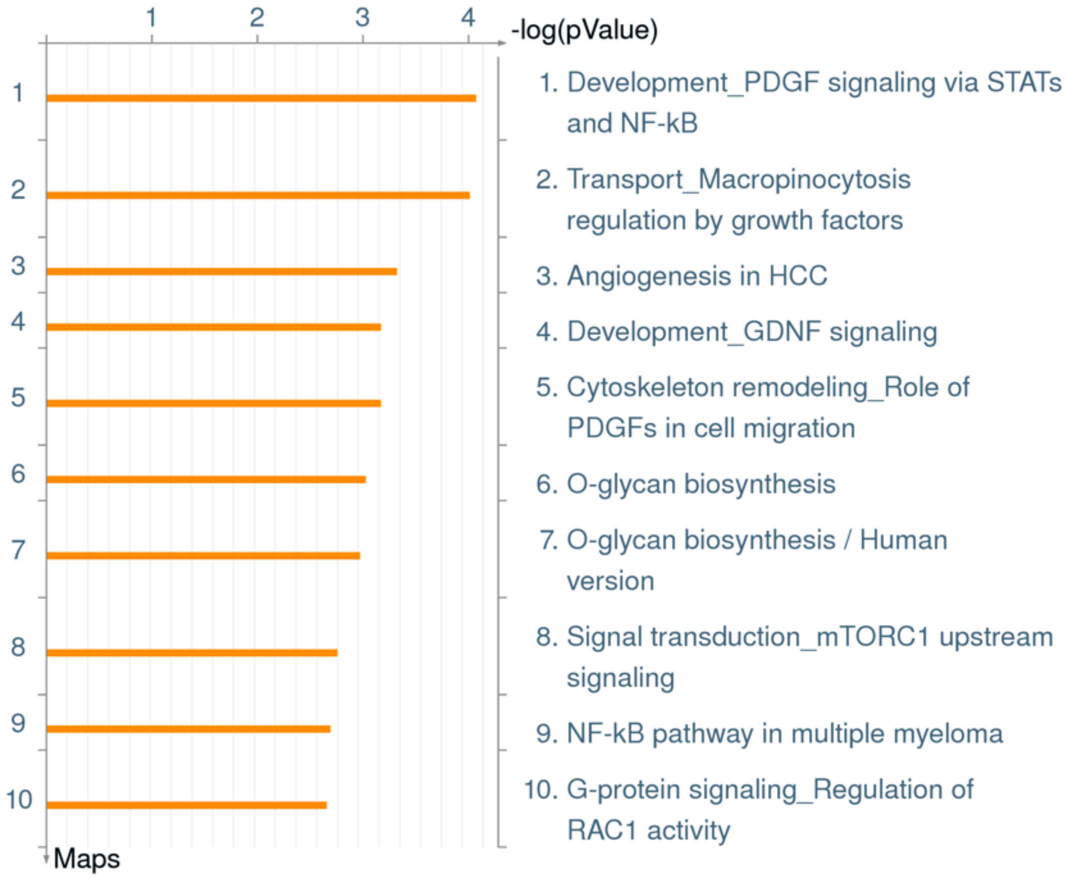

1
2
3
4
-log(pValue)
1
2
3
4
5
6
7
8
9
10
Maps
1. Development_PDGF signaling via STATs and NF-kB
2. Transport_Macropinocytosis regulation by growth factors
3. Angiogenesis in HCC
4. Development_GDNF signaling
5. Cytoskeleton remodeling_Role of PDGFs in cell migration
6. O-glycan biosynthesis
7. O-glycan biosynthesis / Human version
8. Signal transduction_mTORC1 upstream signaling
9. NF-kB pathway in multiple myeloma
10. G-protein signaling_Regulation of RAC1 activity

Figure S2

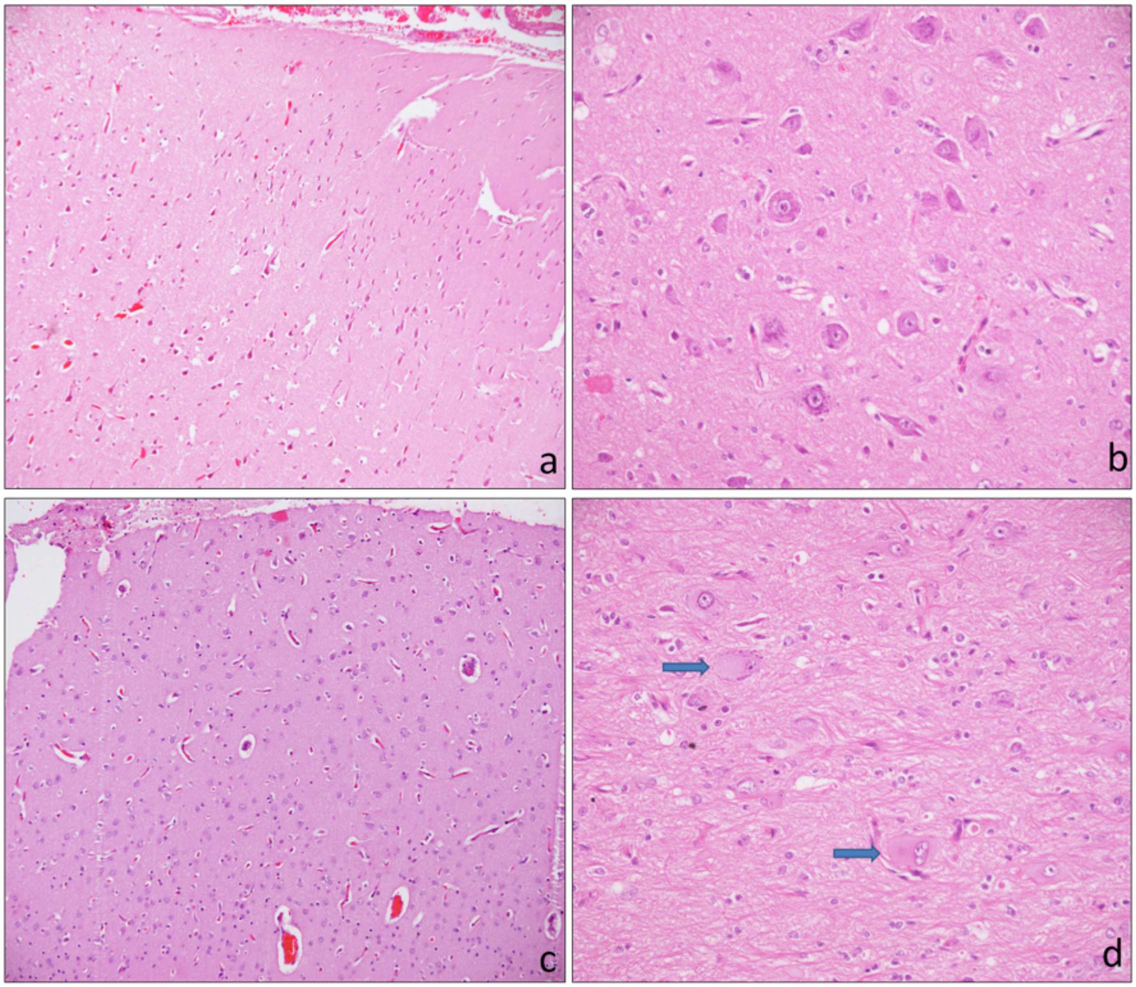

Figure S3

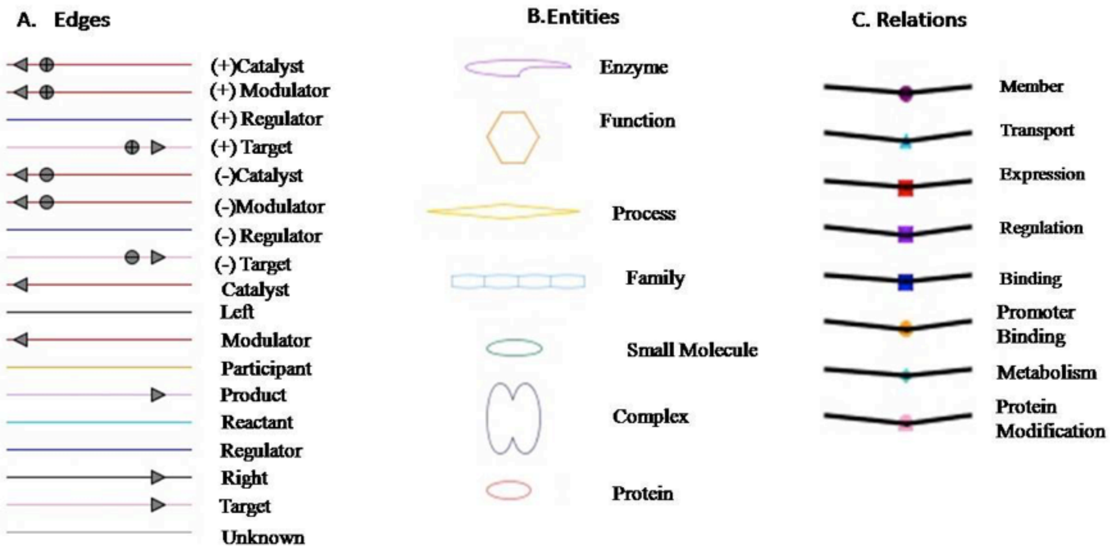
A. Edges
(+)Catalyst
(+) Modulator
(+) Regulator
(+) Target
(-)Catalyst
(-)Modulator
(-) Regulator
(-) Target
Catalyst
Left
Modulator
Participant
Product
Reactant
Regulator
Right
Target
Unknown
B.Entities
Enzyme
Function
Process
Family
Small Molecule
Complex
Protein
C. Relations
Member
Transport
Expression
Regulation
Binding
Promoter Binding
Metabolism
Protein Modification
